# New anatomical information of the wukongopterid *Kunpengopterus sinensis* Wang et al., 2010 based on a new specimen

**DOI:** 10.7717/peerj.4102

**Published:** 2017-12-01

**Authors:** Xin Cheng, Shunxing Jiang, Xiaolin Wang, Alexander W.A. Kellner

**Affiliations:** 1 Key Laboratory of Vertebrate Evolution and Human Origins of Chinese Academy of Sciences, Institute of Vertebrate Paleontology and Paleoanthropology, Beijing, China; 2 Laboratory of Systematics and Taphonomy of Fossil Vertebrates, Department of Geology and Paleontology, National Museum/UFRJ, Rio de Janeiro, Brazil; 3 University of Chinese Academy of Sciences, Beijing, China

**Keywords:** *Kunpengopterus sinensis*, Wukongopteridae, Pterosauria, Yanliao Biota, Late Jurassic, China

## Abstract

The Wukongopteridae compose a non-pterodactyloid clade of pterosaurs that are the most abundant flying reptiles in the deposits of the Middle-Late Jurassic Yanliao Biota. Until now, five species of three genera and two additional unnamed specimens have been described. Here we report on a new material, IVPP V 23674, that can be referred to the wukongopterid *Kunpengopterus sinensis* due to several features such as a comparably short nasoantorbital fenestra, the dorsally rising posterodorsal margin of the ischium, and the very short first pedal phalanx of digit V relative to metatarsal IV. IVPP V 23674 provides the first view of a wukongopterid palate, which differs from all other pterosaurs by having a very large postpalatine fenestra and laterally compressed choanae, indicating that the evolution of the pterosaur palate was more complex than previously thought. Sesamoid bones at the dorsal side of manual unguals are present and are reported for the first time in a wukongopterid suggesting an arboreal life-style for these pterosaurs.

## Introduction

Pterosaurs are the first vertebrates to achieve powered flight. These flying reptiles developed a comparatively fragile skeleton that resulted in a generally limited preservation potential (e.g., Wellnhofer, 1991; Kellner, 1994). As a consequence, except for three so far monotaxic bonebeds (Chiappe et al., 2000; Wang et al., 2014a; Manzig et al., 2014), most species are represented only by one or two specimens (e.g., Wang et al., 2007; Wang et al., 2014b; Jiang & Wang, 2011; Cheng et al., 2015).

In the last ten years, important new pterosaur discoveries have been made in several regions of China, mostly coming from the Late Jurassic Tiaojishan Formation and the Early Cretaceous Jehol Group (e.g., Wang et al., 2009; Wang et al., 2012; Wang et al., 2014b; Wang et al., 2015; Lü et al., 2010; Lü et al., 2011a; Lü et al., 2011b; Cheng et al., 2012; Cheng et al., 2015; Cheng et al., 2017; Jiang et al., 2014; Jiang et al., 2016; Rodrigues et al., 2015). Among the most exciting discoveries done recently are the Wukongopteridae, whose members combine characters from both, basal non-pterodactyloids and derived pterodactyloids (e.g., Wang et al., 2009; Lü et al., 2010; Cheng et al., 2016). So far, six specimens of this non-pterodactyloid clade have been described and referred to three genera and five species (Wang et al., 2009; Wang et al., 2010; Lü et al., 2010; Lü et al., 2011a). In addition, there is *Changchengopterus pani*, known from two individuals (Lü, 2009; Zhou & Schoch, 2011), that is regarded as a potential wukongopterid (Wang et al., 2009; Cheng et al., 2017), and another three undetermined specimens (Lü et al., 2011b; Cheng et al., 2016; Cheng et al., 2017). Furthermore, there are several undescribed fossils scattered throughout many collections in China that most certainly belong to this clade.

Here we report on a second specimen (IVPP V 23674) of *Kunpengopterus sinensis* that provides supplementary anatomical information for this genus, including details of the foot and caudal vertebrae, allowing us to revise the diagnosis of this species. This material also makes it possible to describe, for the first time, the palate of a wukongopterid that reveals several significant differences from other pterosaurs.

## Materials and Methods

IVPP V 23674 consists of a nearly complete skeleton with the skull exposed in right lateral view. It was recovered from the Yanliao deposits near Daxishan (Linglongta, Jianchang, Liaoning Province, China). The specimen was collected by a local resident and is presently housed at the Institute of Vertebrate Paleontology and Paleoanthropology, Chinese Academy of Sciences (IVPP/CAS). It arrived at the IVPP divided into several parts that were put together using epoxy glue and was prepared mechanically by steel needle and pneumatic micro tools under a microscope.

Photographs were taken using camera Nikon D3X and combined into a fully focused image using Helicon Focus 6.7.1 software.

## Results

### Systematic paleontology

**Table:** 

Pterosauria Kaup, 1834
Wukongopteridae Wang et al., 2009
*Kunpengopterus* Wang et al., 2010
*Kunpengopterus sinensis* Wang et al., 2010

Holotype

IVPP V 16047, an almost complete skeleton with the complete skull and lower jaw housed at the Institute of Vertebrate Paleontology and Paleoanthropology (Chinese Academy of Sciences), Beijing, China.

Referred specimen

**Figure 1:**
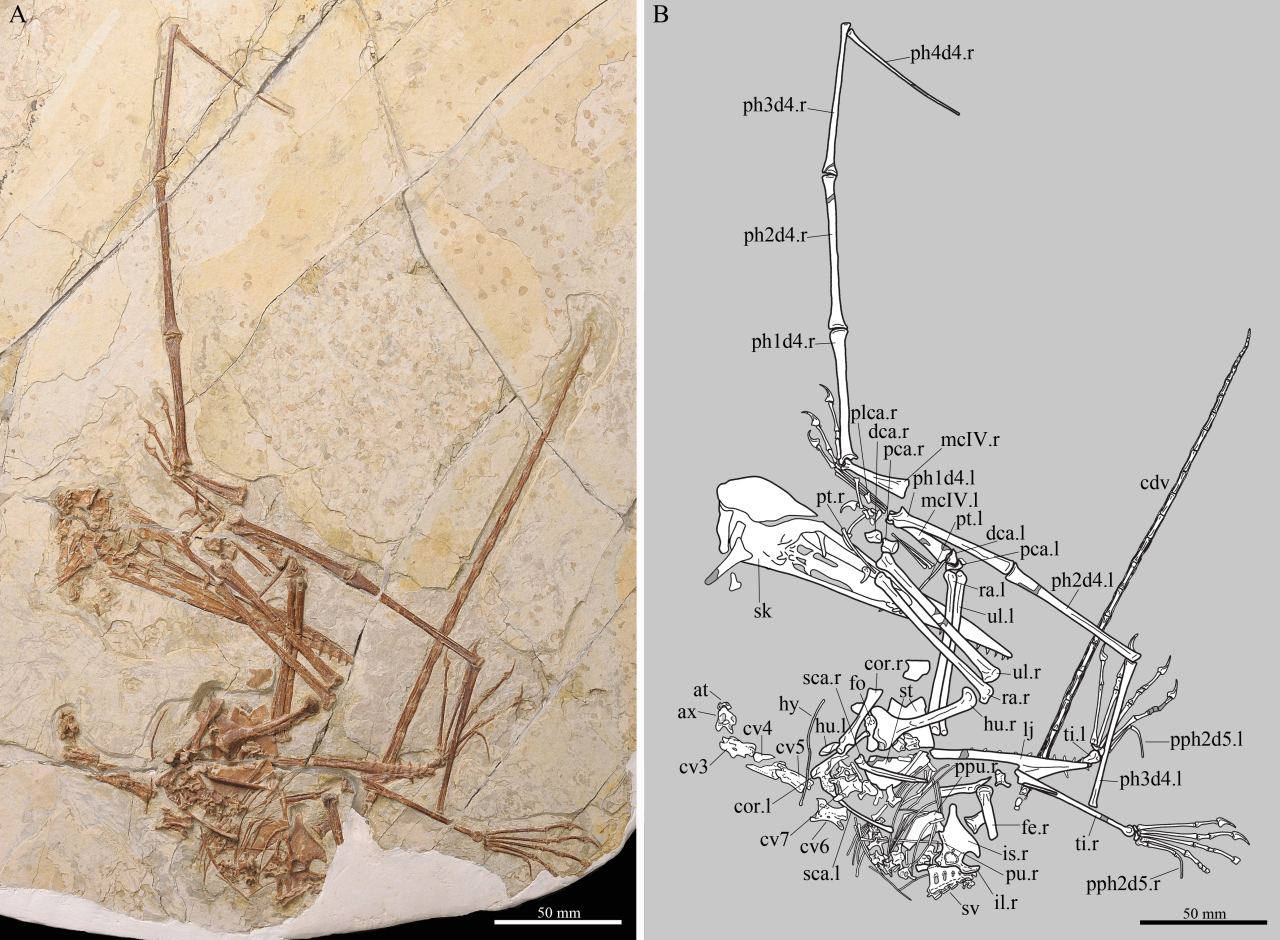
The complete skeleton of *Kunpengopterus sinensis* (IVPP V 23674). (A) Photo and (B) line drawing with missing portions of the skeleton were preserved as impression in dark grey. Scale bars: 50 mm. Abbreviations: at, atlas; ax, axis; cdv, caudal vertebra; cor, coracoid; cv3–7, third to seventh cervical vertebra; dca, distal carpal series; fe, femur; fo, foramen; hu, humerus; hy, hyoid bone; il, ilium; is, ischium; l, left; mcIV, metacarpal IV; pca, proximal carpal series; ph1–4d4, first to fourth phalange of manual digit IV; plca, proximal lateral carpal; ppu, prepubis; ptd, pteroid; r, right; pu, pubis; ra, radius; sca, scapula; st, sternum; sv, sacral vertebra; ti, tibia; ul, ulna.

A nearly complete skeleton including the skull and lower jaw (IVPP V 23674), housed at the IVPP (Fig. 1).

Emended diagnosis

Wukongopterid pterosaur with the following combination of characters that distinguishes it from other members of this clade (autapomorphies are marked with an asterisk): posterior region of the skull rounded*; absence of premaxillary crest; nasoantorbital fenestra around 40% the skull length*; maxillary process of the jugal thin and relatively short*; lacrimal process of the jugal thick; presence of a soft tissue crest above the frontal; posterodorsal margin of ischium rising dorsally; proximal end of prepubis very short and wide*; first pedal phalanx of digit V short, less than 70% of metatarsal IV*; curved second pedal phalanx of the fifth toe with an angle between the proximal and distal segments about 145°*; proximal segment of the second pedal phalanx of the fifth toe about 30% length of the distal segment, shorter than in other wukongopterids* (modified from Wang et al., 2010).

Locality and horizon

Daxishan, Linglongta, Jianchang, Liaoning, China. Tiaojishan Formation (= Daohugou Bed) Late Jurassic (Zhou, Jin & Wang, 2010; Cheng, 2013; Wang et al., 2014c; Sullivan et al., 2014).

**Figure 2:**
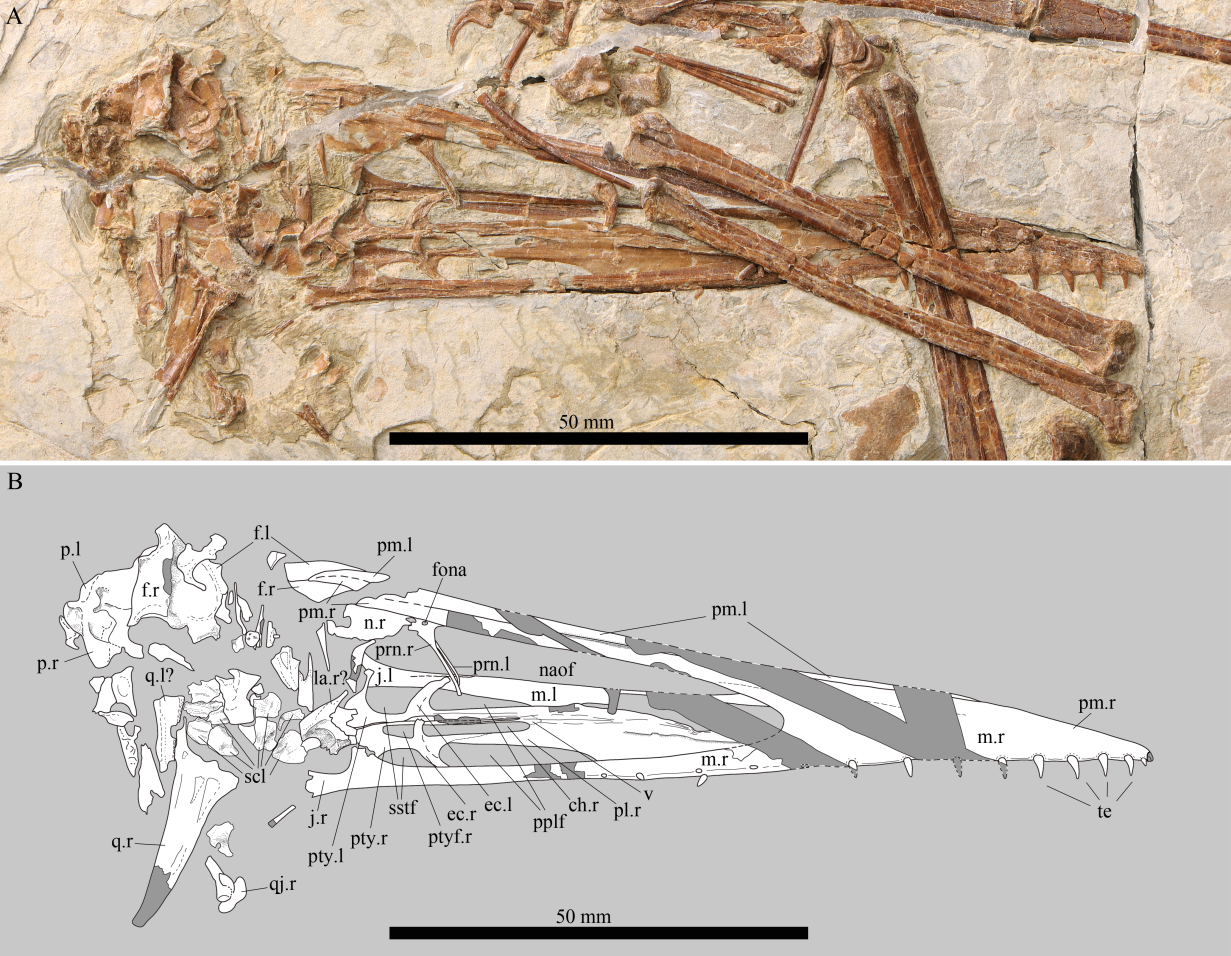
Details of the skull of *Kunpengopterus sinensis* (IVPP V 23674). (A) Close up of the right lateral view. (B) Line drawing, with missing parts and regions covered by other skeletal elements in dark grey. Scale bars: 50 mm. Abbreviations: ch, choana; ec, ectopterygoid; f, frontal; fona, foramen nasale; j, jugal; l, left; la, lacrimal; m, maxilla; n, nasal; naof, nasoantorbital fenestra; pl, palatine; pm, premaxilla; pplf, postpalatine fenestra; prn, process of nasal; pty, pterygoid; ptyf, pterygoid fenestra; q, quadrate; qj, quadratojugal; scl, sclerotic ring; sstf, secondary subtemporal fenestra; te, teeth; v, vomer; ?, uncertain.

Description and comparison

IVPP V 23674 is preserved in a grey-white slab of shale associated with many conchostracans that indicate a freshwater lacustrine environment (Fig. 1). It is composed of a nearly complete skeleton only lacking the tip of the left wing and most of both femora. The skull and lower jaws are complete but disarticulated, with the posterior portion compacted and covered by other bones. Most of the remaining portion of the skeleton is present, with elements closely associated or in articulation (e.g., the right wing, tibiae and feet). The close association of the bones indicates that they represent one individual.

As in the holotype, the skull is elongated and lacks a premaxillary crest (Fig. 2), differing from *Darwinopterus* (Wang et al., 2010; Lü et al., 2010; Lü et al., 2011a) and IVPP V 17959 (Cheng et al., 2016). The length of the nasoantorbital fenestra relative to the skull length is the shortest among all wukongopterids (∼40.1%, Table 1; Cheng et al., 2017). Unfortunately, this region in the holotype (IVPP V16047) is not complete, but based on the preserved portion, the maximum length of this opening relative to the skull length is estimated as being very similar to IVPP V 23674 (∼41%, Table 1).

The premaxilla and maxilla are fused, and no suture is visible (Fig. 2). The posterior end of the premaxillae separates the anterior end of the frontals. On the dorsal margin of the premaxillae, a low bony ridge is present that does not form a premaxillary crest, differing in this respect from *Darwinopterus* and other wukongopterid specimens (Wang et al., 2010; Lü et al., 2010; Lü et al., 2011a; Cheng et al., 2016; Cheng et al., 2017).

As in the holotype, the maxilla process of the jugal is thin and short (Fig. 2). It occupies about 25% the length of the ventral margin of the nasoantorbital fenestra, being much shorter than in *Darwinopterus linglongtaensis* where this ratio is about 50%. The lacrimal process of the jugal is wide and differs from the thinner condition of this bone in *Darwinopterus linglongtaensis* and IVPP V 17959 (Wang et al., 2010; Cheng et al., 2016).

**Figure 3:**
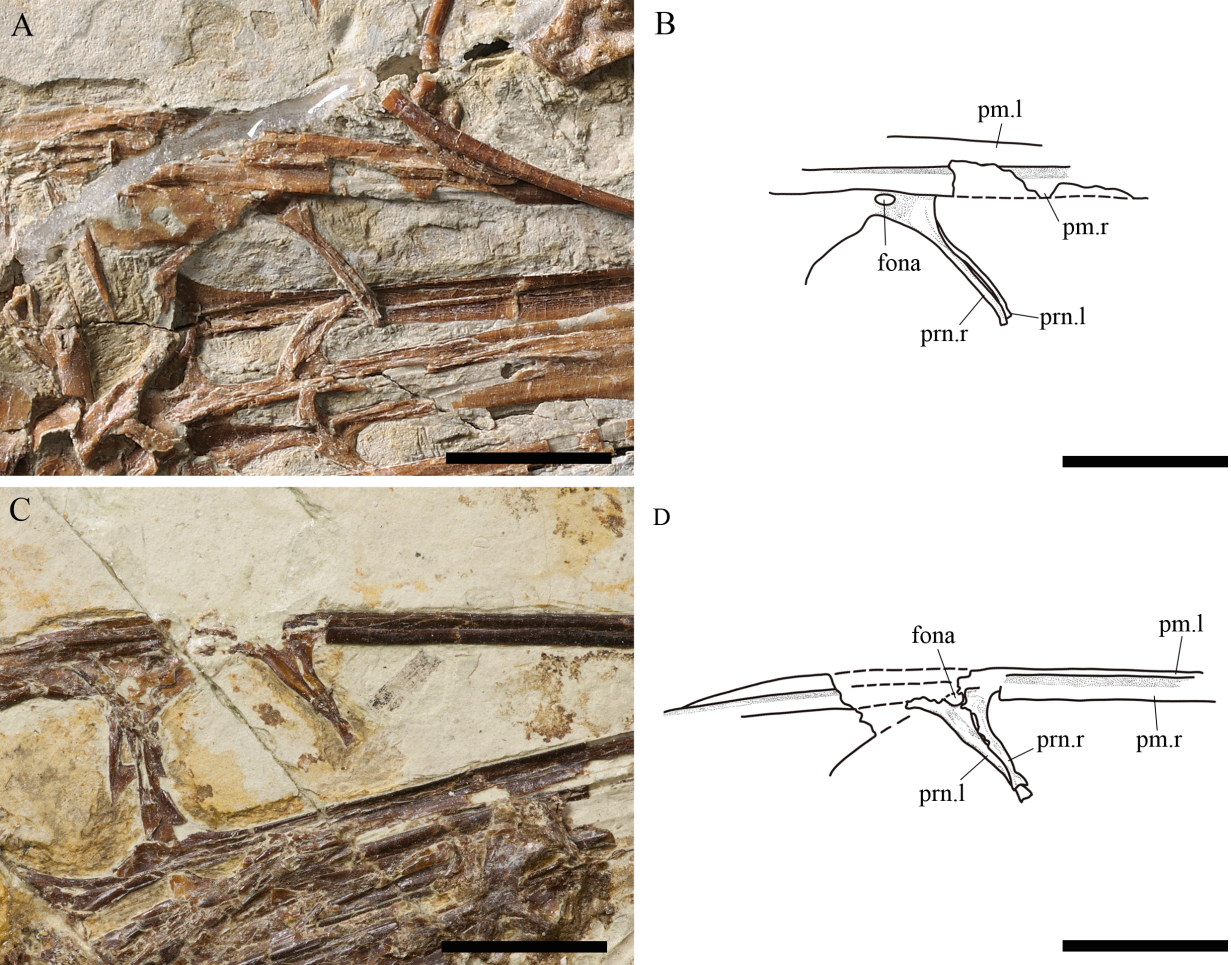
Comparison between the nasal processes of both specimens of *Kunpengopterus sinensis*; IVPP V 23674 (referred specimen) and IVPP V 16047 (holotype). Close up (A) and line drawing (B) of the nasal processes of IVPP V 23674. Close up (C) and line drawing (D) of the nasal processes of IVPP V 16047. Scale bars: 10 mm. Abbreviations: fona, foramen nasale; l, left; pm, premaxilla; prn, process of nasal; r, right.

The nasal bears a thin anteroventrally inclined process (Figs. 2 and 3), which is like the one of *Darwinopterus* (Wang et al., 2010; Lü et al., 2010; Lü et al., 2011a) and different from the subvertical process present in IVPP V 17959 (Cheng et al., 2016). The holotype of *Kunpengopterus sinensis* (IVPP V16047) was originally described as having a broad nasal process, with an elliptical foramen at the middle portion (Wang et al., 2010). However, based on our reexamination of this specimen, the “broad” condition is the result of the overlap of the nasal processes from both sides. There is a foramen positioned at the base of this process.

**Table 1:** Measurements (in mm) of two specimens of *Kunpengopterus sinensis*: IVPP V 23674 (referred specimen) and IVPP V 16047 (holotype).

Bones	IVPP V 23674	IVPP V 16047	IVPP V 23674 / IVPP V 16047
sq-pm	∼120.0	106.9	∼112%
ros	43.8	∼40.5	∼108%
naof	48.8	max 43.9^a^	min 111%
man.sys	27.9	–	
sca	33.0(l)	∼28.8(r)	∼115%
cor	28.9(r)		
	28.3(l)	∼23.8(l)	∼119%
hu	42.8(r)	39.6^a^(r)	108%
ul	66.2(r)		
	64.5(l)	∼59.2(l)	∼109%
mcIV	26.9(r)		
	27.6(l)	∼23.0(l)	∼120%
ph1d4	52.8(r)		
	52.8(l)	∼54.2(r)	∼97%
ph2d4	58.6(r)		
	58.5(l)	58.0(r)	101%
ph3d4	59.1(r)		
	59.0(l)	59.2(r)	100%
ph4d4	53.9(r)		∼110%
		∼48.8(l)	
ti	56.1(r)		103%
		54.5(l)	
mt1	22.6(r)	22.4^a^(r)	101%
	22.9(l)	21.6^a^(l)	106%
mt2	23.1(r)	23.2^a^(r)	100%
	23.2(l)	22.1^a^(l)	105%
mt3	21.6(r)	21.8(r)	99%
	20.8(l)	20.7(l)	100%
mt4	18.8(r)	17.9(r)	104%
	17.9(l)		
pph1d5	11.8(r)	11.2(r)	106%
	12.2(l)		
	this paper	(Wang et al., 2010) and this paper	

**Notes.**

a New values based on the reexamination of IVPP V 16047.

Abbreviations cor coracoid hu humerus l left man.sys mandibular symphysis max maximum mcIV metacarpal IV min minimum mt1–4 metatarsal I–IV naof nasoantorbital ph1–4d4 first to fourth phalanx of manual digit IV pm premaxilla pph1d5 first phalanx of pedal digit V r right ros rostrum sca scapula sq squamsal ti tibia ul ulna

**Figure 4:**
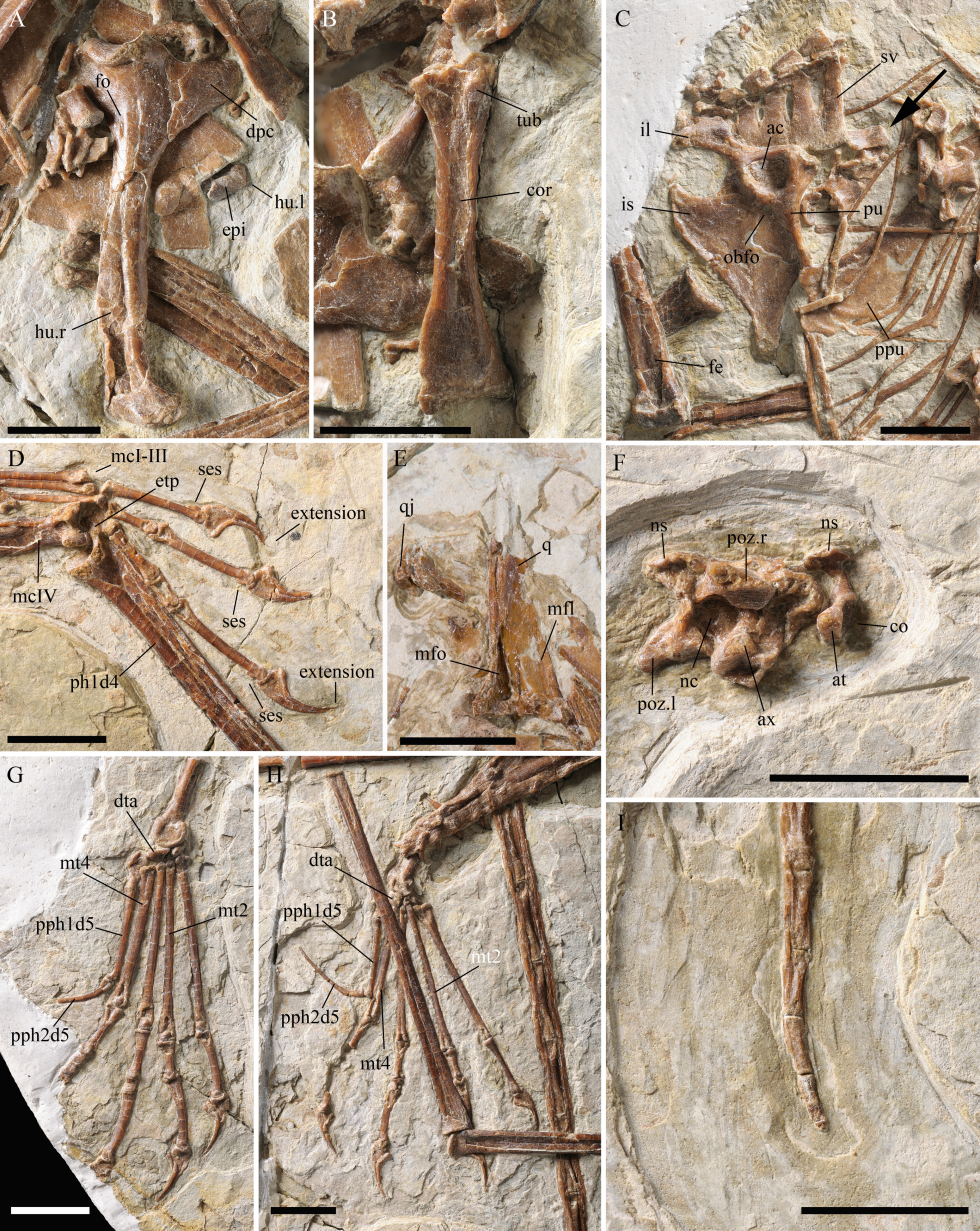
Details of the skeleton of *Kunpengopterus sinensis* (IVPP V 23674). (A) Close up of the right humerus. (B) Close up of the right coracoid. (C) Close up of the sacral vertebrae, pelvic girdle and prepubis, with the arrow pointing to the anterior tip of the right ilium. (D) Close up of right manual digit I–III, with sesamoid bones preserved at the dorsoposterior region of the unguals. (E) Close up of the quadrate. (F) Close up of the atlas and axis. (G) Close up of the right foot. (H) Close up of the left foot. (I) Close up of the distal end of tail. Scale bars: 10 mm. Abbreviations: ac, acetabulum; at, atlas; ax, axis; co, cotyle; cor, coracoid; dpc, deltopectoral crest; dta, distal tarsal; epi, epiphysis; etp, extensor tendon process; fe, femur; fo, foramen; hu, humerus; il, ilium; is, ischium; l, left; obfo, obturator foramen; mcI–IV, metacarpal I–IV; mfl, medial flange; mfo, medial fossa; mt2, metatarsal II; mt4, metatarsal IV; nc, neural canal; ns, neural spine; ph1d4, first phalanx of manual digit IV; poz, postzygapophysis; pph1d5, first phalanx of pedal digit V; pph2d5, second phalanx of pedal digit V; ppu, prepubis; pu, pubis; q, quadrate; qj, quadratojugal; r, right; ses, sesamoid bone; sv, sacral vertebra; tub, tubercle. The names of palate structures are following Wellnhofer (1978) and Kellner (2013).

**Figure 5:**
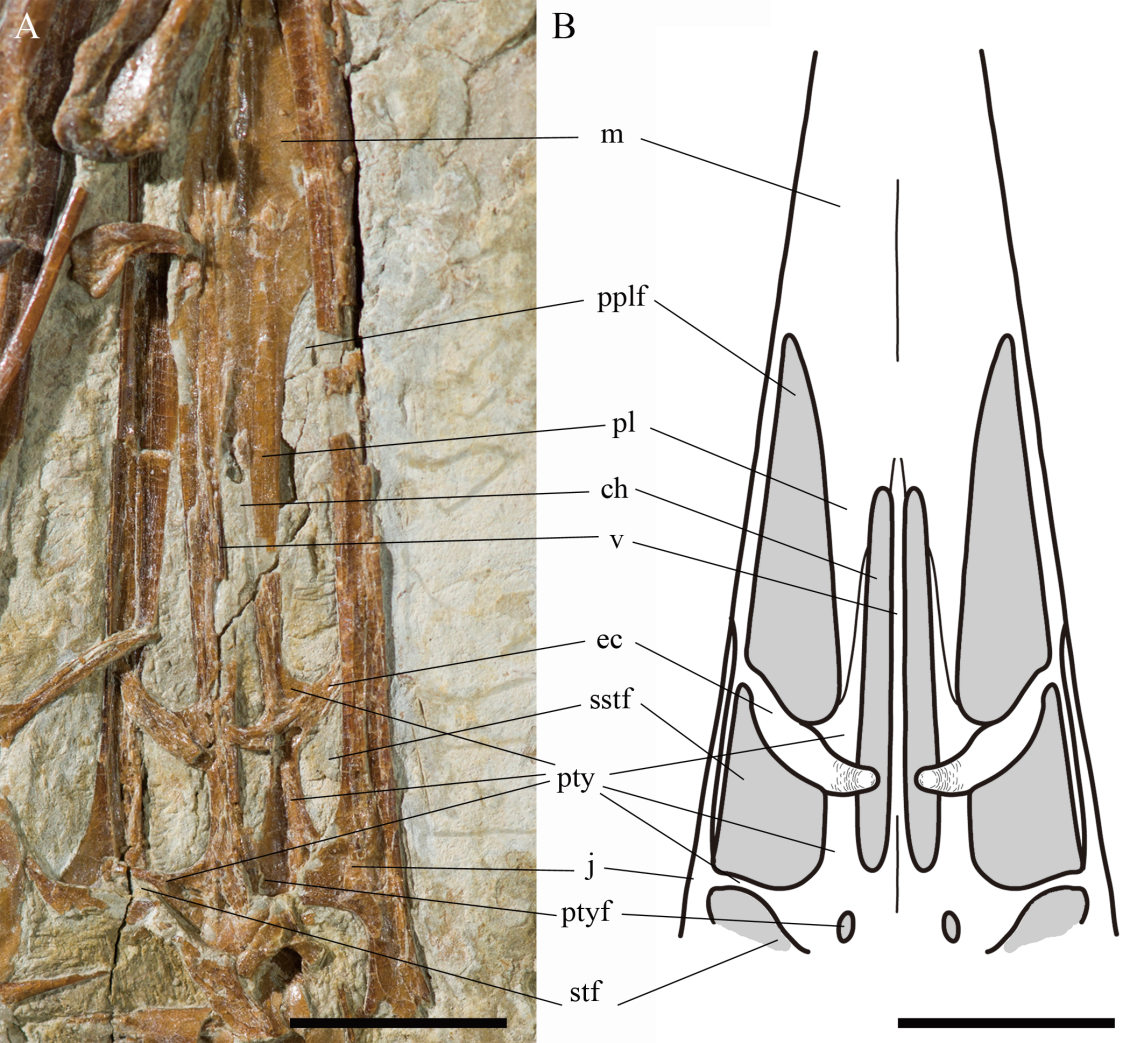
Reconstruction of the partial palate region of *Kunpengopterus sinensis* (IVPP V 23674) in dorsal view. (A) Close up of the palate. (B) Reconstruction. Scale bars: 10 mm. Abbreviations: ch, choana; ec, ectopterygoid; j, jugal; m, maxilla; pl, palatine; pplf, postpalatine fenestra; pty, pterygoid; ptyf, pterygoid fenestra; sstf, secondary subtemporal fenestra; stf, subtemporal fenestra; v, vomer. The names of palate structures are following Wellnhofer (1978) and Kellner (2013).

The posterior region of the skull suffered from severe compression. Several elements have been displaced from their original anatomical position and are broken, making their identification difficult. The shape of the orbit cannot be established. There are about ten small bony plates that made part of the sclerotic ring. An elongated bone displaced ventrally is interpreted as the lacrimal, showing a large opening, similar to the one reported in IVPP V 17959 (Cheng et al., 2016). The quadrate is also displaced and exposed in medial view (Fig. 2). It shows the ventral portion expanded, has a developed medial flange, and shows a deep medial fossa above the articulation (Fig. 4E).

The palate is exposed in dorsal view through the nasoantorbital fenestra with the anterior and posterior portions embedded in the matrix (Figs. 2 and 5). The choanae are elongated (length of the right one - 18.9 mm) and laterally compressed, divided by a thin vomer. The postpalatine fenestrae are extremely elongated, more than in any non-pterodactyloid. They are pear-shaped with the maximum length around 19 mm (preserved length: 18.8 mm) and maximum estimated width around 3.5 mm (preserved width: 3.4 mm). Each is followed by a secondary subtemporal fenestra, which has an overall quadrangular shape (Figs. 2 and 5). Only the anterior margin of the left subtemporal fenestra, made by a lateral extension of the pterygoid, is observed. There is a tiny opening between the pterygoids that appears to be the pterygoid fenestra.

No clear distinction between the ventral part of the maxillae and the palatines is perceptible. The ectopterygoids are compressed and have a posterior ascending process. They form the posterior margin of the postpalatine fenestrae. The pterygoids show a complex morphology and participate in every fenestra of the palate.

The lower jaw is exposed in ventral view (Fig. 1). Its exact length cannot be determined since the posterior end of the mandibular ramus from both sides is covered by other elements of the skeleton. The dentaries are fused forming an elongated mandibular symphysis (Table 1). The preserved angle between both mandibular rami is about 14°.

This new specimen has the dentition better preserved than that of the holotype. Teeth are cone-shaped, with at least fifteen upper and eleven lower teeth. Of these, seven are located on the mandibular symphysis. The last preserved upper tooth is positioned nearly underneath the middle portion of the nasoantorbital fenestra. The interalveolar space of the upper teeth becomes larger posteriorly until the last preserved alveolus that is closer to the preceding one compared to the others.

The atlas and axis are not fused (Fig. 4F). The atlas can be observed from the left laterodorsal view and shows the anterior articulation surface more concave than in IVPP V 17959 (Cheng et al., 2016). Only the neural arch from the left side is preserved and is dorsally expanded. The axis bears large postzygapophyses and a developed neural spine. The centrum is biconvex, with the condyle more developed. The lateral side is concave and is perforated by a small foramen.

Cervical vertebrae 3 to 7 are elongated compared to other non-pterodactyloids (Fig. 1; Table 2) that is a typical feature of the Wukongopteridae (Wang et al., 2009; Wang et al., 2010; Lü et al., 2010; Lü et al., 2011a; Cheng et al., 2016). A lateral depression can be observed on the centrum of cervical 3 and 6, but it is not sure if it leads to a foramen. Several cervical vertebrae show small cervical ribs.

**Table 2:** Measurements of cervical vertebrae of *Kunpengopterus sinensis* (IVPP V 23674) (in mm).

Cervical vertebrae	Length
atlas	1.2
axis	6.8
3rd	11.5
4th	∼13.3
5th	13.0
6th	12.1
7th	8.6

At least 11 dorsal vertebrae, mostly disarticulated, could be identified and do not form a notarium. Some show a foramen on the ventral side of the transverse process. Five sacral vertebrae, all fused, are present with most of the left side broken away (Fig. 4C).

The tail shows at least twenty-six articulated caudal vertebrae (Figs. 1, 4I). Except for the last one, all are enclosed by rod-like extensions of the zygapophyses and chevrons, a typical feature of non-pterodactyloids. The sixth preserved caudal vertebra is the longest, and all after this one decrease gradually in size (Table 3). No trace of a tail vane is preserved.

**Table 3:** Measurement of caudal vertebrae of *Kunpengopterus sinensis* (IVPP V 23674) (in mm). The measurements follow the sequence of the preserved elements. The tail is incomplete and the first caudal vertebrae are not preserved.

Preserved vertebrae	Length	Preserved vertebrae	Length	Preserved vertebrae	Length
1	–	10	11.54	19	7.14
2	3.15	11	11.26	20	6.46
3	–	12	10.84	21	5.48
4	–	13	10.24	22	4.69
5	11.86	14	9.61	23	3.83
6	12.59	15	9.11	24	3.45
7	12.17	16	8.88	25	2.91
8	12.01	17	8.66	26	0.55
9	11.88	18	7.69		

**Notes.**

– measurement not possible due to preservation

The sternum is exposed in dorsal view and is partially covered by the right scapula, coracoid and humerus (Figs. 1, 4A, 4B). This bone is heart-shaped, being longer (40.4 mm) than wide (28.3 mm). The articular facets for the coracoids are asymmetrical and positioned laterally, with the right one in front of the left. A large and rounded foramen is present posterior to the cristospine.

Scapula and coracoid from both sides are preserved, with the former longer than the latter (Table 1). They are fused forming a scapulocoracoid (Fig. 1). The scapula has an elongated and dorsoventrally compressed posterior end. The coracoid bears a well-developed lateral tubercle positioned close to the coracoid process (Fig. 4B), which is also seen in IVPP V 17959 (Cheng et al., 2016). The articulation surface of the coracoid with the sternum is concave and lacks a posterior expansion (Fig. 4B).

Both humeri are preserved, with the left one covered by other skeletal elements. The right humerus is exposed in dorsolateral view, has a slightly curved shaft and shows a foramen on the dorsal surface at the level of the distal margin of the deltopectoral crest (Fig. 4A), similar to that of *Darwinopterus linglongtaensis* (Wang et al., 2010). The deltopectoral crest is placed proximally and expands distally, without a neck or the hatch-shaped condition seen in *Rhamphorhynchus* (Wellnhofer, 1978; Kellner, 2003). The distal end of the left humerus is exposed in ventral view and apparently has the epiphysis fused (Fig. 4A). The ulnae and radii are well preserved, with the diameter of the radius smaller but larger than half that of the ulna (Fig. 1).

The carpal region of both sides is well preserved, with all elements of the proximal and distal carpal series fused. The right distal carpal series can be observed from distal view and shows a trapezoidal outline, with the posterior portion larger than the anterior one (Fig. 1).

The right metacarpal region is complete. Metacarpals I–III are all about the same length and show approximately the same thickness. The pteroid is elongated and slightly curved.

Manual digits I–III bear curved and sharp unguals, which are of similar size as the pedal unguals. All show a well-developed lateral groove. A sesamoid bone is present at the dorsal side of each right manual ungual (Fig. 4D). Examining the holotype (IVPP V 16047), sesamoid bones were also found in the same region that have been overlocked in the original description. At the tip of two manual unguals of IVPP V 23674, there is a small dorsally curved extension that appear to be pathologic (Fig. 4D).

Both first wing phalanges are exposed in dorsal view and have the extensor tendon process fused (Fig. 4D). The first wing phalanx is slightly bent posteriorly, which is a common feature within the Wukongopteridae (Cheng et al., 2016). The length of the first and fourth wing phalanges are smaller than the second and third (Table 1).

The right pelvic girdle is preserved and exposed in lateral view (Fig. 4C). All elements are fused to each other but not to the sacral vertebrae. The preacetabular process of the ilium was broken away. A small obturator foramen can be observed below the acetabulum. The ischium forms a broad ventral narrowing plate with a rising posterodorsal margin. The same configuration is observed in the holotype (IVPP V 16047) and differs from *Darwinopterus linglongtaensis* (Wang et al., 2010). An unusual and plate-like bone is interpreted as the right prepubis since it is tightly connected to the right pubis. Exposed in dorsal view, this element is wide and slightly concave. The anterior margin is clearly thinner than the posterior one, and the articulation with the opposite prepubis is almost straight. Compared to *Darwinopterus* and IVPP V 17959, the proximal end of this bone is very short and wide (Wang et al., 2010; Lü et al., 2010; Lü et al., 2011a; Cheng et al., 2016).

The hindlimbs are not very well preserved and provide only limited information. The distal end of the fibula is fused with the tibia and a clear suture between these elements is only visible at the proximal part. The proximal tarsal elements are fused to the tibia forming a tibiotarsus (Figs. 4G, 4H).

**Figure 6:**
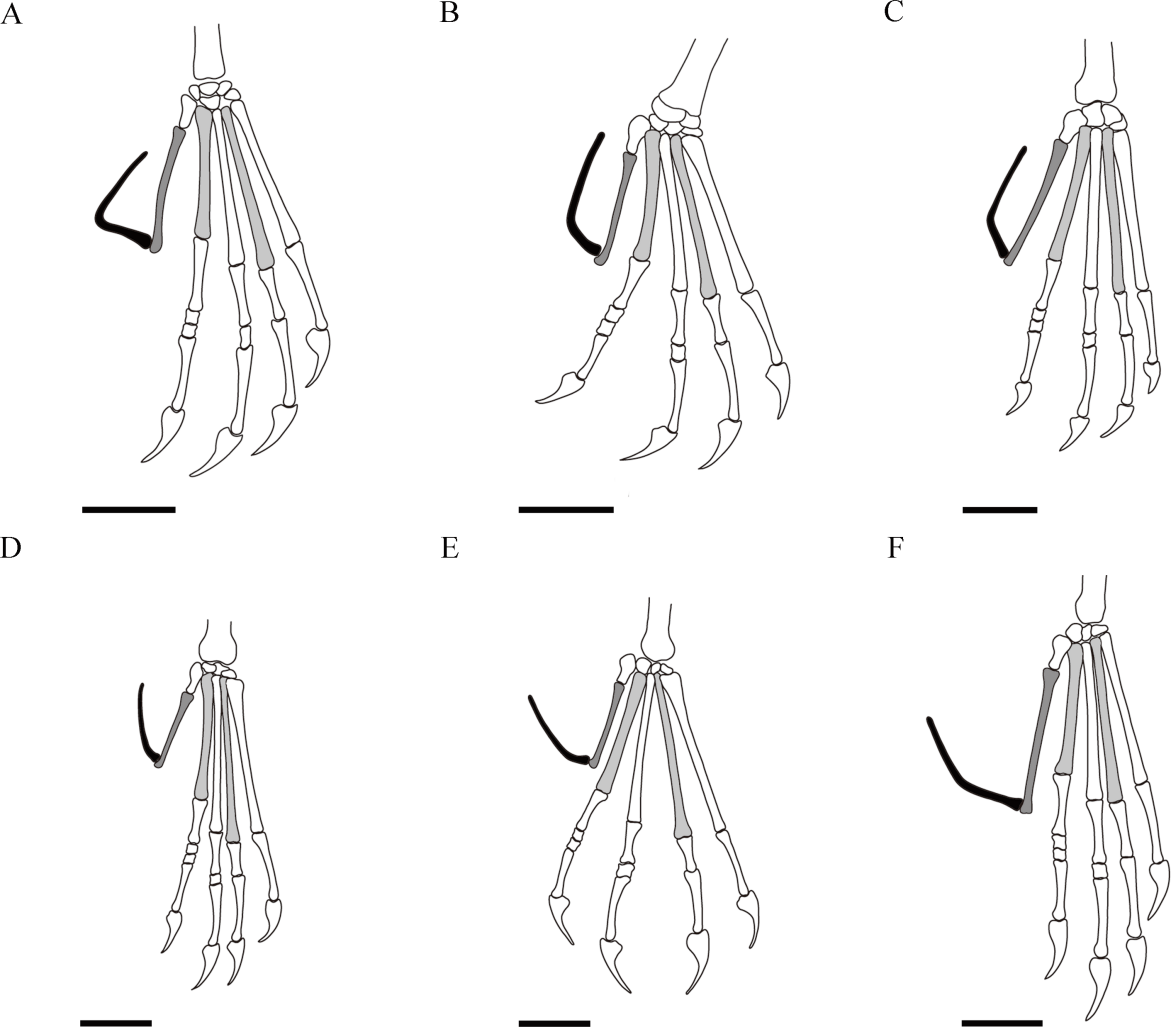
Reconstructions of wukongopterid feet with all metatarsals II of the same size. Showing metatarsals II and IV in light grey, first phalanges of pedal digit V in dark grey and second phalanges in black. Scale bars: 10 mm. (A) *Wukongopterus* (based on Wang et al., 2009). (B) *Darwinopterus linglongtaensis* (based on Wang et al., 2010). (C) *Darwinopterus robustodens* (based on Lü et al., 2011a). (D) Holotype of *Kunpengopterus sinensis* (based on Wang et al., 2010). (E) *Kunpengopterus sinensis* (IVPP V 23674). (F) IVPP V 18403/ZMNH M8802 (wukongopterid with eggs, based on Lü et al., 2011b; Wang et al., 2015).

Both feet are well preserved. Metatarsal II is the longest while metatarsal IV the shortest, with metatarsal I and III of similar length (Tables 1 and 4; Figs. 4G, 4H, 6). The pedal digit V bears two elongated phalanges, with the first one about 21.2% the length of the tibia. The second phalanx is curved near the proximal end, with the proximal segment about 30% the length of the distal one. The angle between the proximal and distal segments is about 145°.

**Table 4:** Measurements (in mm) and ratios of wukongopterid feet.

Taxa	A	B	A/B	C	pph1d5 length	mt2 length	mt4 length	pph1d5/ mt2	pph1d5/ mt4	mt4/ mt2	Reference
*Kunpengopterus sinensis* (IVPP V 16047, holotype)	2.6	8.8	0.30	145°	11.2	23.2	17.9	0.48	0.63	0.77	Wang et al., 2010 and this paper
*Wukongopterus lii*	6.6	9.0	0.73	71°	13.7	18.1	15.0	0.77	0.91	0.83	Wang et al., 2009 and this paper
*Darwinopterus linglongtaensis*	4.5	9.9	0.45	124°	12.1	16.8	14.2	0.72	0.85	0.85	Wang et al., 2010 and this paper
IVPP V 23674	3.0	10.2	0.29	147°	11.8	23.1	18.8	0.51	0.63	0.81	this paper
IVPP V 18043 (wukongopterid with eggs)	^a^7.7	^a^9.7	0.79	^a^137°	18.5	21.0	16.0	0.88	1.16	0.76	Lü et al., 2011b and this paper
*Darwinopterus robustodens*	5.3	11.0	0.48	136°	18.1	22.4	18.5	0.81	0.98	0.83	Cheng et al., 2017 and this paper

**Notes.**

a measurement based on picture.

Abbreviations A length of proximal segment of second phalanx of pedal digit V B length of distal segment of second phalanx of pedal digit V C angle between proximal and distal segment of second phalanx of pedal digit V mt2 metatarsal II mt4 metatarsal IV pph1d5 first phalanx of pedal digit V

## Discussion

IVPP V 23674 can be classified in the Wukongopteridae since it shares several characters with the members of this clade, including a confluent nasoantorbital fenestra, comparatively elongated cervical vertebrae, reduced cervical ribs, and an elongated metacarpal IV (e.g., Wang et al., 2010; Cheng et al., 2017). Wang et al. (2010) also considered the first phalanx of manual digit IV shorter than the fourth as a diagnostic character of this clade, but IVPP V 23674 and at least another two unpublished wukongopterid specimens bear a longer first phalanx of manual digit IV compared to the fourth.

This new specimen (IVPP V 23674) is referred to as *Kunpengopterus sinensis* based on the following features: comparatively short nasoantorbital fenestra relative to the length of the skull, relatively short and thin maxillary process of the jugal, posterodorsal margin of ischium rising dorsally, first pedal phalanx of digit V less than 70% length of metatarsal IV, and second pedal phalanx of the fifth toe curved at an angle of about 145°, with the proximal segment about 30% the length of the distal one. IVPP V 23674 also differs from *Darwinopterus* by the absence of a premaxillary crest and a comparably reduced preacetabular portion of the ilium.

Based on previous studies regarding pterosaur ontogeny (Bennett, 1993; Bennett, 1995; Bennett, 1996; Kellner & Tomida, 2000; Kellner et al., 2013; Kellner, 2015), IVPP V 23674 shows evidence of skeletal maturity. Several bones are fused, including the extensor tendon process with the first phalanx of the wing finger, the tibia with the proximal tarsals, and the scapula with the coracoid (Figs. 4D, 4G, 4H). Based on the six ontogenetic stages (OS1–OS6) introduced by Kellner (2015), IVPP V 23674 has reached at least OS5.

The holotype of *Kunpengopterus sinensis* has also several fused bones (Wang et al., 2010), except for the epiphysis with the distal end of the humerus and the proximal tarsal elements with the tibia. Therefore, from the ontogenetic point of view, the holotype (IVPP V 16047) was younger than the new specimen (IVPP V 23674).

The maximized wingspan (see Kellner et al., 2013) of IVPP V 23674 is about 790 mm. This is roughly 7% larger than the holotype (Table 1; Wang et al., 2010; Cheng et al., 2017).

Pterosaur material with a well-preserved palatal region is not common (e.g., Ősi et al., 2010; Kellner, 2013; Zhou et al., 2017), especially considering non-pterodactyloids (Wellnhofer, 1978). Regarding the Wukongopteridae, despite the several specimens described so far, none has the palate exposed (Wang et al., 2009; Wang et al., 2010; Lü et al., 2010; Lü et al., 2011a; Cheng et al., 2016).

Based on IVPP V 23674, one of the most remarkable palatal features of *Kunpengopterus sinensis* is the very large postpalatine fenestra that reaches almost the same length as the choanae (Figs. 2 and 5), what was never observed in any pterosaur before (e.g., Williston, 1902; Wellnhofer, 1978; Wang et al., 2008; Bennett, 2013; Ősi et al., 2010; Pinheiro & Schultz, 2012; Kellner, 2013). The choanae are reduced, smaller and positioned more posteriorly than the postpalatine fenestrae, which is also observed for the first time in pterosaurs (Figs. 2 and 5).

Ősi et al. (2010) reviewed the palate of pterosaurs and pointed out that the lateral process of the pterygoid that divided the subtemporal fenestra in non-pterodactyloids was lost in pterodactyloids, which was challenged by some recent discoveries (e.g., Kellner, 2013; Zhou et al., 2017). The palate of *Kunpengopterus* (IVPP V 23674) differs remarkably from that of all other pterosaurs and does not conform to the models proposed by Ősi et al. (2010). Although there are indeed three major palatal openings, as in non-pterodactyloids, the postpalatine fenestra (= suborbital fenestra of Ősi et al., 2010) is the largest reported so far in any pterosaur. These openings extend anteriorly surpassing the anterior margin of the choanae. The latter are also very compressed laterally, being even smaller than the postpalatine fenestrae, unlike the condition observed in pterodactyloids and other non-pterodactyloids. The pterygoids also appear to be more united than in non-pterodactyloids, suggesting that the interpterygoid opening, which is not visible in *Kunpengopterus* (IVPP V 23674), might have been smaller in this genus (or in wukongopterids) than in more basal non-pterodactyloids. This odd morphology indicates that the evolution of the palate in pterosaurs was more complex than previously thought. It is possible that the configuration of the palate observed in IVPP V 23674 might be similar to that of other wukongopterids and therefore could potentially provide several new autapomorphies for this quite remarkable clade.

Regarding the sesamoid bones found associated with the manual unguals of *Kunpengopterus*, such elements were also reported in *Darwinopterus linglongtaensis* (IVPP V 16049), an individual that lacks the fusion of proximal and distal carpal elements and was therefore ontogenetically less developed (probably OS3 of Kellner, 2015) than the two specimens of this genus. This suggests that ungual sesamoids appear very early in the ontogenetic development of these pterosaurs and might have been widespread among wukongopterids. The fact that they have not been reported so far might be due to overlooking, poor preservation or loss during preparation.

The presence of sesamoid bones at the dorsal side of manual unguals have been reported in many basal pterosaurs (e.g., Wild, 1978; Wild, 1994; Padian, 1983; Padian, 2008; Dalla Vecchia, 2009). These ossifications were probably connected with the extensor digitorum brevis (Bennett, 2008), whose function has been linked with grasping capabilities, climbing and terrestrial locomotion (Unwin, 1988; Bennett, 1997; Witton, 2015). The presence of a sesamoid is also observed in at least one pedal phalanx of the holotype (IVPP V 16047) of *Kunpengopterus sinensis*. Therefore, arboreal behavior for this pterosaur and other wukongopterids is a strong possibility that cannot be ruled out.

Regarding the feet of other wukongopterids, *Kunpengopterus* shows a shorter first phalanx of pedal digit V that is less than 70% the length of metatarsal IV (Table 4). Furthermore, the second pedal phalanx of digit V is bent very close to the proximal end, with both sections forming an angle of around 145°.

Wang et al. (2015) regarded a specimen (IVPP V 18403/ZMNH M8802) as potentially representing *Kunpengopterus* sp. However, the feet of IVPP V 18403/ZMNH M8802 differ from other wukongopterids by having the first phalanx of pedal digit V longer than metatarsal IV and the second phalanx of pedal digit V curved at about the middle part of this bone, forming an angle of 137° (Table 4). Although the detailed study of this specimen is beyond the scope of this paper, it appears that IVPP V 18403/ZMNH M88 might present a distinct taxon at least at a species level.

## Conclusion

The discovery of a second specimen (IVPP V 23674) of *Kunpengopterus sinensis* provides new information for this species, including anatomy and ontogeny. IVPP V 23674 can be classified in a later ontogenetic stage (at least OS5) than the holotype. It shows that the fusion of ilium and sacral vertebrae has not been completed at this stage. The new specimen sheds light on the wukongopterid palate that differs from all other pterosaurs by the presence of extremely elongated postpalatine fenestrae that reach anterior to the anterior margin of the choanae, and choanae that are very compressed laterally. This indicates that the palate development in pterosaurs was more complex than previously thought.

The present study shows the presence of sesamoid bones at the dorsal side of manual unguals, which are reported for the first time in this non-pterodactyloid clade. Based on the differences between the wukongopterid pes, *Kunpengopterus* differs from others by showing a shorter first phalanx and the shortest proximal segment of the second phalanx of pedal digit V, indicating that the feet are relevant for wukongopterid taxonomy.

## Additional Information and Declarations

### Data Availability

The following information was supplied regarding data availability: The specimen (IVPP V 23674) described in this article is housed at the Institute of Vertebrate Paleontology and Paleoanthropology, Chinese Academy of Sciences.
